# 

**Published:** 1908-06-06

**Authors:** 


CONTENTS.

| | PAGE |
|---|---|
| **The Case for the Tuberculous Child** ... ... ... | 245 |
| **Intermarriage of Cousins**... ... ... ... ... ... ... | 246 |
| **Annotations—** | |
| L'Entente Cordiale Médicale—The Prevention of Crime—Midwives and Cancer ... ... ... ... ... ... ... | 247 |
| **Medical Opinion and Movement** ... ... ... ... | 248 |
| **Hospital Clinics—** | |
| A Case of Carcinoma of the Œsophagus. By Leonard A. Bidwell, F.R.C.S. ... ... ... ... ... ... | 251 |
| The Retina in Cases of Albuminuria. By Leslie Paton, M.B., F.R.C.S. ... ... ... ... ... ... ... ... | 253 |
| **Medicine—** | |
| The Sequelæ of Enteric Fever—I. ... ... ... ... ... | 255 |
| Lung Disease and Unequal Pupils ... ... ... ... ... | 256 |
| Theocin-Sodium Acetate in the Relief of Dropsy... ... ... | 256 |
| **Points in Surgery—** | |
| The Causation of Gastric Ulcer ... ... ... ... ... | 257 |
| The Surgical Significance of Vomiting ... ... ... ... | 258 |
| **Obstetrics—** | |
| Infection of the Upper Urinary Tract during Pregnancy ... | 259 |
| **Mental Diseases—** | |
| Refusal of Food and Forcible Feeding ... ... ... ... ... | 260 |
| **Resident Medical Officers' Department—** | |
| Accident Cases ... ... ... ... ... ... ... ... ... | 261 |
| **Dermatology—** | |
| Pityriasis Rosea ... ... ... ... ... ... ... ... ... | 262 |
| **Pathology—** | |
| Cammidge's Pancreatic Reaction ... ... ... ... ... | 263 |
| **Cases for Diagnosis—** | |
| Solutions to Cases Described on Page 238 ... ... ... ... | 265 |
| **New Appliances and Things Medical** ... ... ... | 266 |
| **The Medical Congress at Budapest** ... ... ... | 266 |
| **The Practitioner's Relaxations and Hobbies—** | |
| Green Plover and Stone-Curlew ... ... ... ... ... ... | 267 |
| News and Coming Events ... ... ... ... ... ... ... | 269 |
| **Nursing Administration—** | |
| The Bedding and Linen Department—VI. ... ... ... ... | 271 |
| **The Graduates' Medical Schools** ... ... ... ... | 272 |
| **Editor's Letter-Box** ... ... ... ... ... ... ... ... | 272 |

